# Nitrogen management under increased atmospheric CO_2_ concentration in cucumber (*Cucumis sativus* L.): ameliorating environmental impacts of fertilization

**DOI:** 10.1038/s41598-021-01882-3

**Published:** 2021-11-16

**Authors:** María Carmen Piñero, Ginés Otálora, Josefa López-Marín, Francisco M. del Amor

**Affiliations:** Department of Crop Production and Agri-Technology, Murcia Institute of Agri-Food Research and Development (IMIDA), C/Mayor s/n, 30150 Murcia, Spain

**Keywords:** Plant sciences, Environmental sciences

## Abstract

In the last years, the atmospheric CO_2_ concentration has increased significantly, and this increase can cause changes in various physiological and biochemical processes of plants. However, the response of plants to elevated CO_2_ concentration (e[CO_2_]) will be different depending on the nitrogen form available and the plant species. Therefore, hydroponic trials on cucumber plants, with two CO_2_ concentrations (400 and 1000 ppm) and two nitrogen sources (NO_3_^−^/NH_4_^+^; 100/0 and 90/10), were conducted. Physiological parameters—such as gas exchange, GS, GOGAT and GDH activities, cation composition, soluble sugar and starch content- were measured. The results showed that when plants were grown with NH_4_^+^ and e[CO_2_], parameters such as photosynthesis rate (A_CO2_), instantaneous water use efficiency (WUEi), the content of NH_4_^+^, Ca^2+^ and Mg^2+^, and the concentration of starch, were higher than in control plants (irrigated with nitrate as sole nitrogen source and ambient CO_2_ concentration (a[CO_2_])). Furthermore, an improvement in N assimilation was observed when the GS/GOGAT pathway was enhanced under these conditions (NH_4_^+^ and e[CO_2_]). Thus, our results contribute to the reduction of the negative environmental impacts of the use of nitrogen fertilizers on this crop, both by reducing nitrogen leakage (eutrophication) and greenhouse gas emissions.

## Introduction

The atmospheric carbon dioxide (CO_2_) concentration is increasing at a faster rate and is projected to reach nearly 1000 µmol mol^−1^ by the end of 2100^1^. Since the current CO_2_ concentration (413 ppm) (NOAA 2020) is still a limiting factor for plant growth, and the optimal concentration is considered between 800–1000 ppm^2^, this increase in CO_2_ concentration could favor the photosynthetic rate and stimulate plant growth and development. However, the response of plants to e[CO_2_] varies depending on other environmental factors^3^, such as the nitrogen form (NO_3_^−^ or NH_4_^+^) available^4^. Authors such as Rubio-Asensio and Bloom^4^ reported that plants irrigated with NH_4_^+^ showed a more positive response to e[CO_2_], than those irrigated with NO_3_^−^, as e[CO_2_] inhibited the assimilation of NO_3_^−^ in the shoots of C3 plants.

Both N forms (NO_3_^−^ and NH_4_^+^) share the same metabolic pathway, called as glutamate pathway, in which enzymes such as glutamate synthase (GOGAT) and glutamine synthase (GS) are involved; hence, this pathway is also identified as the GS/GOGAT cycle. GS is responsible for assimilating NH_4_^+^ as amino acids (glutamine), and GOGAT transforms this glutamine to glutamate^5^. Furthermore, glutamate dehydrogenase (GDH) catalyzes the transformation of α-ketoglutaric acid and NH_3_ into glutamate through the glutamate pathway. Consequently, GOGAT, GS and GDH are considered the principal enzymes of N metabolism in higher plants^5^. As the response of plants to an increase in CO_2_ concentration will be different depending on the source of N supplied and the specie considered, it will be very important to better understand the plant’s preference for different N forms under e[CO_2_], as this knowledge can be used to guide farmers towards the more efficient use of fertilizers. Therefore, because of the high energy cost of production of N fertilizers, and the environmental impact of the nitrate that is not taken up by plants^6^, growers must reduce the N losses and pollution associated to fertilization by using the appropriate ratio of inorganic nitrate under future climate scenarios. The nitrogen fertilization of crops is one of main causes of environmental contamination worldwide, through nitrate leaching and as a net contributor to greenhouse gas emission^7,8^. Cucumber (*Cucumis sativus* L.) is one of the most-cultivated vegetables in the world due to its economic and nutritional benefits^5,9^. However, although many cucumber growth parameters have been studied, such as photosynthesis^10^, nitrogen metabolism^11^, fruit quality^12^, or water use efficiency^13^, the combined effects of CO_2_ and different N forms (NO_3_^−^ and NH_4_^+^) have not been studied. Therefore, this study is the first attempt at understanding how N forms and e[CO_2_] interact in cucumber plants in a climate change scenario. To stimulate the physiological mechanisms affected by these two factors (N forms and e[CO_2_]), cucumber plant seedlings were exposed to different N inputs and e[CO_2_] in a controlled environment. The responses of plants were assessed by measuring the net CO_2_ assimilation, internal CO_2_ concentration, instantaneous water use efficiency, cation concentration, nitrogen-metabolizing enzymes, starch, and soluble sugars.

## Results

### Gas exchange

**Figure 1:**
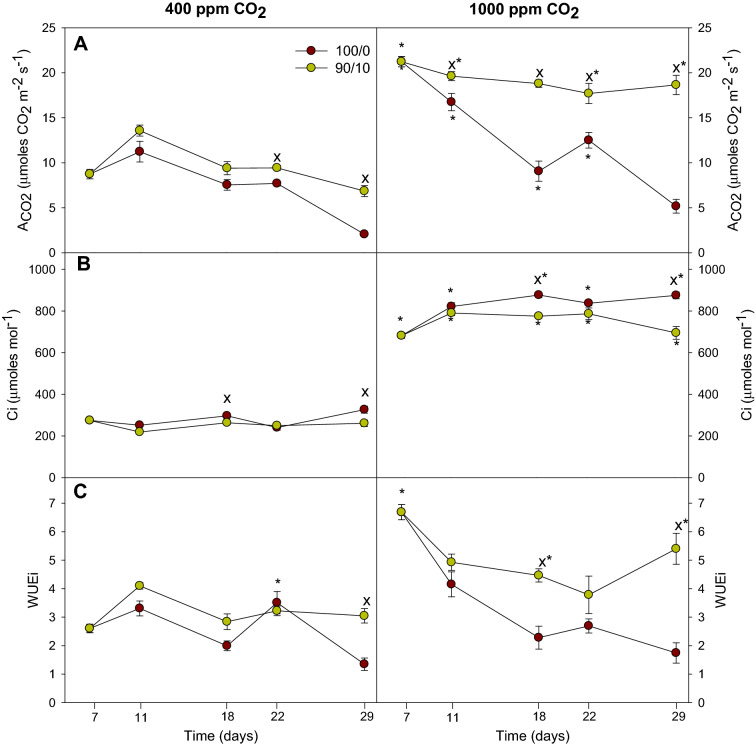
Effect of N forms (100% NO_3_^−^ and 90% NO_3_^−^/10% NH_4_^+^ combined) under an eCO_2_ on cucumber plants: (**A**) net photosynthesis rate (A_CO2_); (**B**) internal CO_2_ concentration (Ci); and (**C**) instantaneous water-use efficiency (WUEi). Data are means ± SE of six plants. * denotes significant differences (*P* < 0.05) between plants in different CO_2_ treatments, with the same nitrogen treatment; X denotes significant differences (*P* < 0.05) between nitrogen treatments for the same CO_2_ treatment.

The data showed that the treatment with NH_4_^+^ (90/10) increased the A_CO2_ at both CO_2_ concentrations (Fig. 1A). The A_CO2_ throughout the experiment slightly decreased at both CO_2_ concentrations. Curiously, the decrease was more pronounced when the plants were irrigated with the 100/0 treatment at both CO_2_ concentrations, but mainly under e[CO_2_]. This decrease was from 21.2 µmoles CO_2_ m^−2^ s^−1^ to 5.2 µmoles CO_2_ m^−2^ s^−1^ in plants irrigated with 100/0 under e[CO_2_]. In contrast, the decrease in plants irrigated with the 100/0 treatment under a[CO_2_] was only from 8.7 µmoles CO_2_ m^−2^ s^−1^ on 7 DAT, to 2.1 µmoles CO_2_ m^−2^ s^−1^ on 29 DAT. On the other hand, exposure to e[CO_2_] markedly increased A_CO2_ in both N treatments (Fig. 1A). Similarly, the Ci increased by CO_2_ enrichment (Fig. 1B). However, this parameter obtained higher values in plants irrigated with the 100/0 treatment at both CO_2_ concentrations, on 18 DAT and 29 DAT. During the rest of the experiment, no significant differences were observed between the irrigation treatments.

With regard to the WUEi, the levels were higher in plants irrigated with the 90/10 treatment under e[CO_2_] than under a[CO_2_] (Fig. 1C). The 90/10 treatment also caused a slight increase of this parameter with respect to plants irrigated with NO_3_^-^ as the sole N source, but with differences significant only on 29 DAT at a[CO_2_] (56%) and on 18 and 29 DAT at e[CO_2_] (49% and 68%, respectively).

### Mineral concentration

The mineral composition was affected by CO_2_ and N form (Table 1). The leaf NH_4_^+^ and Ca^2+^ concentrations were higher at e[CO_2_] than at a[CO_2_] in both N treatments, with the only exception found in the NH_4_^+^ concentration of plants irrigated with the 100/0 treatment, which was reduced (33%). Also, the addition of NH_4_^+^ to the irrigation solution caused an increase in the concentration of Ca^2+^ and Mg^2+^ at both CO_2_ concentrations. But in the case of NH_4_^+^ concentration, this increase was only observed under e[CO_2_] (15%). In contrast, leaf K^+^ concentration was not affected by neither CO_2_ nor N form (Table 1).

**Table 1:** Effect of N forms (100% NO_3_^−^ and 90% NO_3_^−^/10% NH_4_^+^ combined) under an elevated CO_2_ concentration on the cation composition of leaves of cucumber.

[CO_2_]	Nitrogen form	NH_4_^+^ (mmol Kg^−1^)	Ca^2+^ (mmol Kg^−1^)	K^+^ (mmol Kg^−1^)	Mg^2+^ (mmol Kg^−1^)
400	100/0	5.68 ± 0.08^b^	6.21 ± 0.38^d^	118.5 ± 9.8^a^	19.39 ± 2.46^c^
	90/10	5.92 ± 0.04^b^	13.70 ± 0.40^b^	120.4 ± 4.4^a^	32.44 ± 2.38^ab^
1000	100/0	3.83 ± 0.30^c^	9.41 ± 1.13^c^	133.2 ± 5.1^a^	25.53 ± 3.47^bc^
	90/10	7.00 ± 0.27^a^	17.98 ± 0.61^a^	123.7 ± 2.9^a^	34.26 ± 1.63^a^
**ANOVA**^**b**^					
CO_2_		ns	***	ns	ns
NF^c^		***	***	ns	***
CO_2_ × NF		***	ns	ns	ns

^a^Different letters within a column indicate significant (P ≤ 0.05) differences between treatments.

^b^Analysis of variance: ns, not significant.

^c^Nitrogen form.

*P ≤ 0.05.

**P ≤ 0.005.

***P ≤ 0.001.

### Soluble sugars and starch

**Figure 2:**
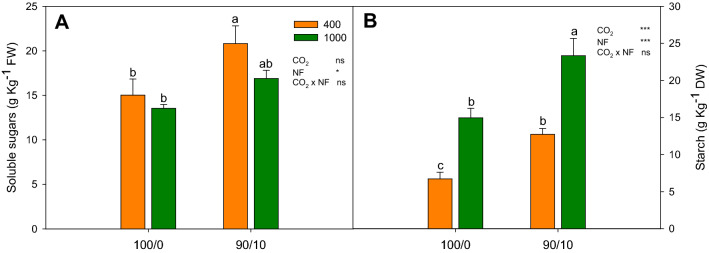
Effect of N forms (100% NO_3_^−^ and 90% NO_3_^−^/10% NH_4_^+^ combined) under an elevated CO_2_ concentration on leaves of cucumber: (**A**) soluble sugars; and (**B**) starch. Data are means ± SE of six plants. Different letters indicate significant (*P* ≤ 0.05) differences between treatments.

The soluble sugars and starch contents were affected by N form (Fig. 2A and B). The sugar content increased mainly under the 90/10 treatment at a[CO_2_] (from 15 g Kg^−1^ FW to 21 g Kg^−1^ FW), while at e[CO_2_], this increase was not significant (Fig. 2A). The starch content rose under both CO_2_ concentrations by the combined application of NO_3_^-^ and NH_4_^+^ (from 7 g Kg^−1^ DW to 13 g Kg^−1^ DW under a[CO_2_] and from 15 g Kg^−1^ DW to 23 g Kg^−1^ DW under e[CO_2_]) (Fig. 2B). On the other hand, the e[CO_2_] provoked an increase in the starch content, from 7 g Kg^−1^ DW to 15 g Kg^−1^ DW in plants irrigated with 100/0 and from 13 g Kg^−1^ DW to 23 g Kg^−1^ DW, in plants irrigated with 90/10 (Fig. 2B).

### Nitrogen-metabolizing enzymes

**Figure 3:**
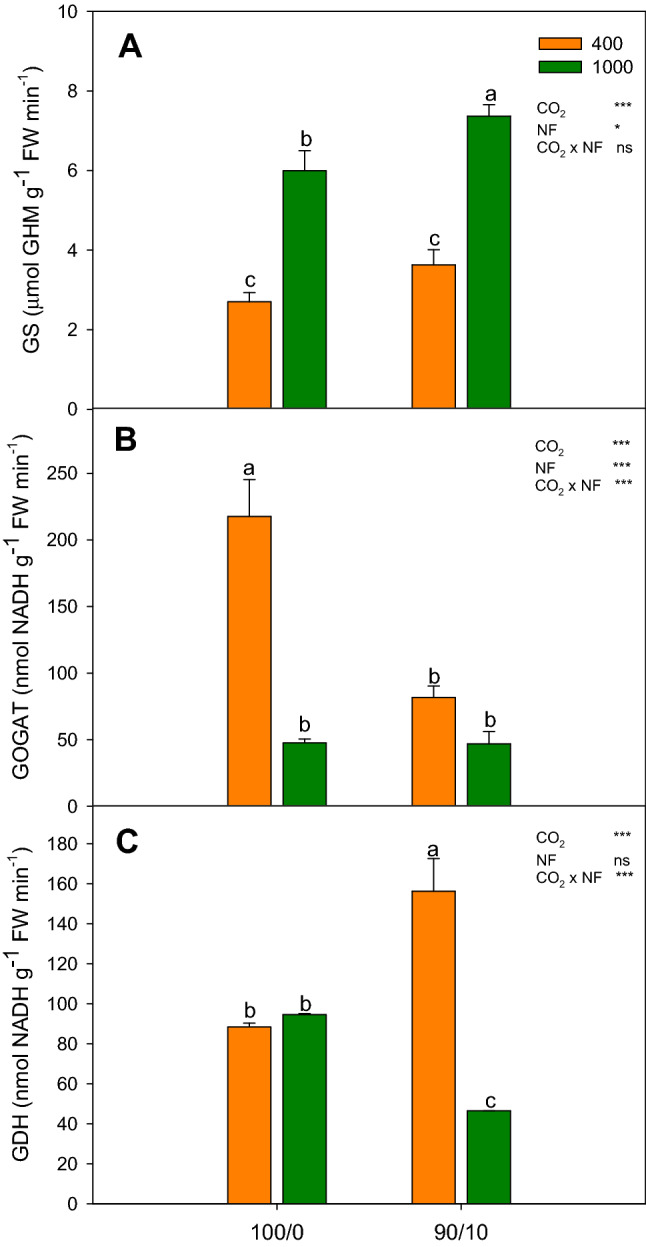
Effect of N forms (100% NO_3_^−^ and 90% NO_3_^−^/10% NH_4_^+^ combined) under an elevated CO_2_ concentration on leaves of cucumber: (**A**) glutamine synthetase activity (GS); (**B**) glutamate synthase activity (GOGAT); and (**C**) glutamate dehydrogenase activity (GDH). Data are means ± SE of six plants. Data are means ± SE of six plants. Different letters indicate significant (*P* ≤ 0.05) differences between treatments.

The behaviors of these 3 enzymes (GS, GOGAT, and GDH) against the N source were dependent on the environmental conditions in which the plants were grown (Fig. 3). Plants grown under a[CO_2_] and irrigated with the 90/10 treatment, suffered a significant reduction in GOGAT activity (62%), and an increase in GDH activity (43%) (Fig. 3B and C). On the contrary, when plants were grown under e[CO_2_], the treatment with a mixture of N forms caused a slight increase in GS activity (19%) and a reduction in GDH activity (51%) (Fig. 3A and C).

## Discussion

The effects on plants of the N form(s) used for irrigation depend on several factors such as environmental conditions (temperature, light intensity, atmospheric CO_2_ concentration, N concentration, average pH, and K supply), the proportions in which they are supplied (NO_3_^−^/NH_4_^+^), the plant species, and even on the variety. Therefore, studies carried out by various authors about the use of different N sources have shown different results^14–16^.

Our data showed that the combination of different N forms (NO_3_^−^/NH_4_^+^) and e[CO_2_] provoked a significant increase in the gas exchange parameters. In our previous studies, we observed a similar behavior in pepper plants exposed to similar conditions^17,18^. Something similar was reported by authors such as Cruz et al*.*^19^ and Bloom et al*.*^20^, who observed that plants (cassava and wheat plants, respectively) exposed to e[CO_2_] and NH_4_^+^-based nutrition showed a greater stimulation of photosynthesis than those irrigated with NO_3_^−^ alone. These results indicates that adding NH_4_^+^ to the nutrient solution increases the photosynthetic capacity of plants grown under e[CO_2_]. This could be due to the energy cost involved in assimilating NO_3_^−^ versus NH_4_^+^: the reduction of NO_3_^−^ to NH_4_^+^ implies the consumption of approximately 10 ATP molecules, while in the conversion of NH_4_^+^ to glutamate, the consumption is reduced to only approximately 2 ATP^4^. Therefore, plants fed with NO_3_^-^ as the N source must efficiently divide and distribute the reducer generated in photosynthesis to cover the additional assimilation demands of NO_3_^−^ assimilation^4^. In the case of WUEi, Cruz et al*.*^19^ just as in the present study, observed the highest WUEi values under e[CO_2_] and NH_4_^+^ nutrition. However, authors such as Torralbo et al*.*^21^ found the opposite effect on photosynthesis in durum wheat, but a similar behavior in WUEi.

Under e[CO_2_] conditions, the foliar NH_4_^+^ concentration results were different depending on the N source supplied to the roots. This is in agreement with the results found by Rubio-Asensio and Bloom^4^, who observed that CO_2_ enrichment inhibited NO_3_^−^ assimilation in plants grown with NO_3_^-^ nutrition, but did not affect those grown with NH_4_^+^ nutrition. This would explain the lower NH_4_^+^ concentration observed in plants treated with 100/0 under e[CO_2_]. In these plants, as NH_4_^+^ is not provided in the nutrient solution, the foliar NH_4_^+^ concentration observed came from the assimilation of NO_3_^-^, which changes from NO_3_^-^ to nitrite (NO_2_^−^) and next, to NH_4_^+^^22^. This suggests that futures increase in atmospheric CO_2_ concentrations may compromise the productivity of some plants if we not change the fertilization strategies.

Curiously, other nutrients such as Ca^2+^ and Mg^2+^ increased in concentration with the 90/10 treatments at both CO_2_ concentrations. However, authors such Boschiero et al*.*^23^ reported contrary effects in sugarcane plants, which showed a reduction in the leaf nutrients with the increase in the NO_3_^−^/NH_4_^+^ ratio. The fact that the 90/10 treatment produced an increase in the foliar Ca^2+^ concentration, despite the antagonistic effect that exists between these two elements^18^, could indicate that at a low concentration of NH_4_^+^, this antagonistic effect does not occur for cucumber plants. A significant positive correlation was found between foliar Ca^2+^ content and WUEi (R^2^ = 0.6708, data no shown). It is known that Ca^2+^ plays an important role in multiple photosynthetic pathways, it can both interfere with gas exchange by regulating stomatal movement, and can directly or indirectly regulate the activity of enzymes involved in photosynthesis. Authors such as Brestic et al*.*^24^ observed that Ca^2+^ improved Rubisco activity, and the higher activity seemed to be associated with a higher photosynthetic rate.

Concerning the values of K^+^, the finding that no differences were observed in foliar K^+^ concentration between treatments, could be another indication that the NH_4_^+^ concentration provided was not toxic for cucumber plants, as K^+^ deficiencies have been observed in toxic concentrations due to competition in absorption between K^+^ and NH_4_^+^^23^. Regarding the CO_2_ effect on the foliar nutrient content, curiously the cucumber plants responded in the opposite manner to the pepper plants under similar growth conditions^18^, which reinforces the idea of significant species-dependent-response.

Authors such as Teng et al.^25^, Markelz et al*.*^26^ and Rubio-Asensio and Bloom^4^, observed that e[CO_2_] provoked an increase in the photosynthesis rate of *Arabidopsis*, which was related with a rise in starch and the total non-structural carbohydrates. In our experiment, something similar was observed with the starch, but no changes were observed in the soluble sugars. This could be due to the increase in starch or other carbohydrates storage polymers being greater than the increase in sugar concentrations, but the extent of the changes vary considerably between species^27^. Also, it is known that the N supplied can have an influence on the accumulation of starch and soluble sugars under e[CO_2_]^27^. In our results, a relationship was once more observed between the increase caused by NH_4_^+^ in photosynthesis and the higher leaf starch content observed.

Regarding the behavior observed in the metabolic enzymes measured, our data showed that the treatment with NH_4_^+^ under an atmosphere enriched with CO_2_ provoked an increase in the GS activity and a reduction in the GDH activity, which indicate that the combination of the 90/10 treatment and e[CO_2_] may promote the GS/GOGAT pathway of N metabolism. This increase in the GS/GOGAT pathway could be partly responsible for a higher photosynthesis rate, as it would favor N assimilation^5^. On the contrary, the treatment with NH_4_^+^ under a[CO_2_] conditions resulted in a higher GDH activity and lesser GOGAT activity than the 100/0 treatment. These results suggest that GDH played a decisive role when the GS/GOGAT pathway was restricted. Authors such as Ma et al*.*^5^ and Torralbo et al*.*^21^ consider that the role of GDH in N metabolism becomes more important when plants are subjected to stress, specifically NH_4_^+^ toxicity. GDH removes excess NH_4_^+^ and thus reduces its toxic effect.

To achieve a better understanding of the trends and relationships among all the studied parameters in relation to the N supply, a PCA was applied to the results. The results of the PCA are presented in Table 2 and presents a clearer distinction of the effects of nitrogen form and CO_2_ concentration. The first two principal components (PCs) accounted for 71.38% of the total variance, attributing 42.26% to PC1 and 29.13% to PC2 (Table 2). Most of the variables examined were positively correlated with PC1, and only two variables were negative correlated with PC1. The variables with the highest positive correlation coefficients were Ca^2+^ (0.925) and WUEi (0.877), and others with a high correlation were soluble sugars (0.685), Mg^2+^ (0.559), A_CO2_ (0.772) and NH_4_^+^ (0.780). PC1 was negatively correlated with GDH (− 0.402) and K^+^ (0.762), allowed for a clear separation of plants irrigated with NH_4_^+^ in the nutrient solution, and suggested that plant growth with the 90/10 treatment was characterized by a higher Ca^2+^ (Table 1), and higher WUEi (Fig. 1C). PC2 was positively correlated with GS (0.871), starch (0.690) and Ci (0.944), and was negatively correlated with GOGAT activity (− 0.669). The PC2 clearly separated plants grown under e[CO_2_], characterized by a higher GS activity (Fig. 2A), and higher Ci (Fig. 1B).

**Table 2:** Eigenvalues, proportion of variation, accumulated proportion of variation, and eigenvectors associated with the two axes of the PCA.

Principal components	1	2
Eigenvalues	5.493	3.786
Proportion of variation	42.256	29.125
Accumulated proportion of variation	42.256	71.381

Characters	Eigenvectors	
A_CO2_	0.772	
WUEi	0.877	
GDH activity	− 0.402	
NH_4_^+^	0.780	
Ca^2+^	0.925	
K^+^	− 0.762	
Mg^2+^	0.925	
Soluble sugars	0.685	
Ci		0.944
GS activity		0.871
GOGAT activity		− 0.669
Starch		0.690

The data obtained in this experiment highlight the complexity and importance of using the correct type of nitrogen fertilization in the plant irrigation solution to face the environmental changes that are currently taking place (increase in CO_2_). We have demonstrated that physiological parameters such as the A_CO2_ and WUEi can be improved in cucumber plants with the addition of NH_4_^+^ in low amounts in the nutrient solution under a CO_2_-enriched atmosphere. Also, under these conditions (NH_4_^+^ and e[CO_2_]), the GS/GOGAT cycle is promoted, which favors the assimilation of N, and the increase in the concentrations of other nutrients such as NH_4_^+^, Ca^2+^, and Mg^2+^, and the starch content.

Consequently, this study reveals the strong interaction between the N form supplied and e[CO_2_], in terms of N assimilation, and therefore, of a better performance of the photosynthetic apparatus.

## Material and methods

### Plant material, growth conditions and treatments

Cucumber (*Cucumis sativus* L.), cv. Ashley seeds (Semillas Batlle, S.A., Barcelona, Spain) were germinated on a mixture of peat and perlite (3:1). Seedlings with two true leaves stages were selected for uniformity after the 12 days, and transplanted to 8-L black containers filled with coconut coir fiber (Pelemix, Alhama de Murcia, Murcia, Spain). Each container was rinsed with 1 L of water after transplanting. Irrigation was supplied by self-compensating drippers (2 lh^−1^), and fresh nutrient solution was applied with a minimum of 35% drainage.

The plant growth responses to different nitrogen forms and e[CO_2_] were determined in an experiment carried out in a climate chamber designed by our department specifically for plant research proposals^28^, with fully-controlled environmental conditions: 30% relative humidity, 16/8 h day/night photoperiod, an air temperature ranging from 28 to 20 °C, and a photosynthetically-active radiation (PAR) of 250 µmol m^−2^ s^−1^ provided by a combination of fluorescent lamps (TL-D Master reflex 830 and 840, Koninklijke Philips Electronics N.V., the Netherlands) and high-pressure sodium lamps (Son-T Agro, Philips). During the first seven days after transplanting (7 DAT), the plants were irrigated with Hoagland’s solution (control), and then, the plants were irrigated with Hoagland’s solutions that differed in their NO_3_^−^/NH_4_^+^ ratios (in concentration percentages, 100/0 or 90/10) for twenty-two days.

The experiment lasted twenty-nine days and was carried out at standard CO_2_ (400 µmol mol^−1^ CO_2_) (a[CO_2_]), and elevated CO_2_ (1000 µmol mol^−1^ CO_2_) (e[CO_2_]) concentrations, with nine plants per treatment. Thus, four treatments were studied, corresponding to two nutrient solutions and two ambient CO_2_ concentrations.

### Statistical analysis

Data were statistically analyzed using the SPSS 13.0 software package (IBM SPSS Statistics 25.0, Armonk, NY, USA), with an ANOVA and Duncan’s multiple range test (*P* ≤ 0.05) using the treatments as a statistical variable to determine significant differences between means.

### Gas exchange

The gas exchange measurements were performed just before starting the nitrogen treatments (7 DAT), and throughout the experiment (11, 18, 22, and 29 DAT). A CIRAS-2 (PP system, Amesbury, MA, USA) with a PLC6 (U) Automatic Universal Leaf Cuvette, was used to measure the net CO_2_ assimilation (A_CO2_), internal CO_2_ concentration (Ci) and instantaneous water-use efficiency (WUEi, A_CO2_/E). The measurements were conducted on the youngest fully-expanded leaf from each plant. The cuvette provided light (LED) with a photon flux of 1300 µmol m^−2^ s^−1^, 400 or 1000 µmol mol^−1^ CO_2_, 70% relative humidity, and a leaf temperature of 26 °C.

### Ion concentrations

The NH_4_^+^, K^+^, Ca^2+^ and Mg^2+^ ions were extracted from ground leaf lyophilized (1 g) with bi-distilled water, and their concentrations were determined in an ion chromatograph (METROHM 861 Advanced Compact IC; METROHM 838 Advanced Sampler); the column used was a METROHM Metrosep C1 125/4.6 mm.

### Starch and soluble sugars

Soluble sugars were extracted by incubating 30–40 mg of lyophilized leaf tissue twice in 5 mL of 60% ethanol, 30 min each time, at 35 °C. The extract was centrifuged at 3500×g for 10 min at 20 °C, and the two supernatants were combined. Chloroform (5 mL) was added and the mixture shaken before centrifugation at 2700×g for 10 min at 20 °C. The sample was diluted fourfold with absolute ethanol to produce an extract in 80% ethanol for the measurement of soluble sugars according to Buysse and Merckx^29^. The residual material from the extraction with 60% ethanol was hydrolyzed with 3% HCl for 3 h at 125 °C, and the soluble sugars released were measured as an estimate of the starch content^30^.

### Nitrogen-metabolizing enzymes extraction and assay

Fresh leaf tissue samples were frozen with liquid nitrogen and stored at − 20 °C until analysis. Between 0.5 g of plant tissue were pulverized under liquid nitrogen with a chilled pestle and mortar and then homogenized with 5 mL ice-cold enzyme extraction buffer containing 50 mM Tris–HCl, pH 8; 1 mM EDTA, 10 mM β-mercaptoethanol, 5 mM dithiothreitol (DTT), 10 mM MgSO_2_ 7H_2_O, 6.6% of PVPP (polyvinylpolypyrrolidone), 1 mM Cysteine, and 0.5 mM phenylmethylsulfonyl (PMSF).

After centrifugation at 17,000×g at 4 °C for 20 min, the supernatant was collected and used for enzyme assays. The activity of NADH-GOGAT (EC 1.4.1.14.) and GDH (EC 1.4.1.2.) were assayed spectrophotometrically according to Groat and Vance^31^ by monitoring the oxidation of NADH at 340 nm. The activity of glutamine synthetase (GS, EC 6.3.1.2) was assayed spectrophotometrically according to the modified method by Setién et al.^32^, and the absorbance of γ-glutamyl monohydroxamate (γ-GHM) was measured at 540 nm.

## References

[1] Pachauri, R. K. *et al. Ottmar Edenhofer (Germany), Ismail Elgizouli (Sudan), Christopher B. Field (USA), Piers), Mark Howden (Australia)*. *Kristin Seyboth (USA)* (Gian-Kasper Plattner).

[2] Jin C (2009). Carbon dioxide enrichment by composting in greenhouses and its effect on vegetable production. J. Plant Nutr. Soil Sci.

[3] Kimball BA, Kobayashi K, Bindi M (2002). Responses of agricultural crops to free-air CO_2_ enrichment. Adv. Agron..

[4] Rubio-Asensio JS, Bloom AJ (2017). Inorganic nitrogen form: A major player in wheat and *Arabidopsis* responses to elevated CO_2_. J. Exp. Bot..

[5] Ma C (2019). Urea addition promotes the metabolism and utilization of nitrogen in cucumber. Agronomy.

[6] Atkinson D (2005). Prospects, advantages and limitations of future crop production systems dependent upon the management of soil processes. Ann. Appl. Biol..

[7] Hakeem KR, Ahmad A, Iqbal M, Gucel S, Ozturk M (2011). Nitrogen-efficient rice cultivars can reduce nitrate pollution. Environ. Sci. Pollut. Res..

[8] Rodrigues J (2019). Multi-omic and physiologic approach to understand Lotus japonicus response upon exposure to 3, 4 dimethylpyrazole phosphate nitrification inhibitor. Sci. Total Environ..

[9] Zhang Y (2015). Physical and chemical indices of cucumber seedling leaves under dibutyl phthalate stress. Environ. Sci. Pollut. Res..

[10] Dong J (2017). High nitrate supply promotes nitrate assimilation and alleviates photosynthetic acclimation of cucumber plants under elevated CO_2_. Sci. Hortic. (Amsterdam).

[11] Agüera E, Ruano D, Cabello P, de la Haba P (2006). Impact of atmospheric CO2 on growth, photosynthesis and nitrogen metabolism in cucumber (*Cucumis sativus* L.) plants. J. Plant Physiol..

[12] Dong J (2018). Elevated and super-elevated CO_2_ differ in their interactive effects with nitrogen availability on fruit yield and quality of cucumber. J. Sci. Food Agric..

[13] Sánchez-Guerrero MC, Lorenzo P, Medrano E, Baille A, Castilla N (2009). Effects of EC-based irrigation scheduling and CO_2_ enrichment on water use efficiency of a greenhouse cucumber crop. Agric. Water Manag..

[14] Pinero MC, Perez-Jimenez M, Lopez-Marin J, del Amor FM (2016). Changes in the salinity tolerance of sweet pepper plants as affected by nitrogen form and high CO_2_ concentration. J. Plant Physiol..

[15] Imran M (2019). Molybdenum-induced effects on nitrogen metabolism enzymes and elemental profile of winter wheat (*Triticum aestivum* L.) under different nitrogen sources. Int. J. Mol. Sci..

[16] Pedersen IF, Sørensen P, Rasmussen J, Withers PJ, Holton G (2019). Fertilizer ammonium: nitrate ratios determine phosphorus uptake in young maize plants. J. Plant Nutr. Soil Sci..

[17] Piñero MC, Pérez-Jiménez M, López-Marín J, del Amor FM (2016). Changes in the salinity tolerance of sweet pepper plants as affected by nitrogen form and high CO_2_ concentration. J. Plant Physiol..

[18] Piñero MC, Pérez-Jiménez M, López-Marín J, Varó P, del Amor FM (2018). Differential effect of the nitrogen form on the leaf gas exchange, amino acid composition, and antioxidant response of sweet pepper at elevated CO_2_. Plant Growth Regul..

[19] Cruz JL, Alves AAC, Lecain DR, Ellis DD, Morgan JA (2014). Effect of elevated CO_2_ concentration and nitrate: Ammonium ratios on gas exchange and growth of cassava (*Manihot esculenta* Crantz). Plant Soil.

[20] Bloom AJ, Smart DR, Nguyen DT, Searles PS (2002). Nitrogen assimilation and growth of wheat under elevated carbon dioxide. Proc. Natl. Acad. Sci. U. S. A..

[21] Torralbo F, González-Moro MB, Baroja-Fernández E, Aranjuelo I, González-Murua C (2019). Differential regulation of stomatal conductance as a strategy to cope with ammonium fertilizer under ambient versus elevated CO_2_. Front. Plant Sci..

[22] Lin D (2020). Evaluation of seed nitrate assimilation and stimulation of phenolic-linked antioxidant on pentose phosphate pathway and nitrate reduction in three feed-plant species. BMC Plant Biol..

[23] Boschiero BN, Mariano E, Azevedo RA, Ocheuze Trivelin PC (2019). Influence of nitrate: Ammonium ratio on the growth, nutrition, and metabolism of sugarcane. Plant Physiol. Biochem..

[24] Brestic M, Olsovska K, Yang X, Tan W, Wei Meng Q (2011). Photosynthesis is improved by exogenous calcium in heat-stressed tobacco plants. Artic. J. Plant Physiol..

[25] Teng N (2006). Elevated CO_2_ induces physiological, biochemical and structural changes in leaves of *Arabidopsis thaliana*. New Phytol..

[26] Markelz RJC, Vosseller LN, Leakey ADB (2014). Developmental stage specificity of transcriptional, biochemical and CO_2_ efflux responses of leaf dark respiration to growth of *Arabidopsis thaliana* at elevated [CO2]. Plant Cell Environ..

[27] Stitt M, Krapp A (1999). The interaction between elevated carbon dioxide and nitrogen nutrition: The physiological and molecular background. Plant Cell Environ..

[28] del Amor FM, Cuadra-Crespo P, Walker DJ, Cámara JM, Madrid R (2010). Effect of foliar application of antitranspirant on photosynthesis and water relations of pepper plants under different levels of CO_2_ and water stress. J. Plant Physiol..

[29] Buysse J, Merckx R (1993). An improved colorimetric method to quantify sugar content of plant tissue. J. Exp. Bot..

[30] Walker DJ, Romero P, De Hoyos A, Correal E (2008). Seasonal changes in cold tolerance, water relations and accumulation of cations and compatible solutes in *Atriplex halimus* L. Environ. Exp. Bot..

[31] Groat RG, Vance CP (1981). Root nodule enzymes of ammonia assimilation in Alfalfa (*Medicago sativa* L.). Plant Physiol..

[32] Setién I (2013). High irradiance improves ammonium tolerance in wheat plants by increasing N assimilation. J. Plant Physiol..

